# Untargeted Metabolic Profiling Cell-Based Approach of Pulmonary Artery Smooth Muscle Cells in Response to High Glucose and the Effect of the Antioxidant Vitamins D and E

**DOI:** 10.3390/metabo8040087

**Published:** 2018-11-30

**Authors:** Abdulwahab Alamri, Abdulhadi S. Burzangi, Paul Coats, David G. Watson

**Affiliations:** 1 Department of Pharmacology, College of Pharmacy Sciences, University of Hail, 55476 Hail, Saudi Arabia; 2 Strathclyde Institute of Pharmacy and Biomedical Science, University of Strathclyde, 161 Cathedral Street, Glasgow G4 0RE, UK; abdulhadi.burzangi@strath.ac.uk (A.S.B.); paul.coats@strath.ac.uk (P.C.); d.g.watson@strath.ac.uk (D.G.W.)

**Keywords:** oxidative stress, pulmonary arterial hypertension, smooth muscle cells, metabolomics, metabolic profiling, diabetes mellitus, hyperglycaemia, vitamin D, vitamin E

## Abstract

Pulmonary arterial hypertension (PAH) is a multi-factorial disease characterized by the hyperproliferation of pulmonary artery smooth muscle cells (PASMCs). Excessive reactive oxygen species (ROS) formation resulted in alterations of the structure and function of pulmonary arterial walls, leading to right ventricular failure and death. Diabetes mellitus has not yet been implicated in pulmonary hypertension. However, recently, variable studies have shown that diabetes is correlated with pulmonary hypertension pathobiology, which could participate in the modification of pulmonary artery muscles. The metabolomic changes in PASMCs were studied in response to 25 mM of D-glucose (high glucose, or HG) in order to establish a diabetic-like condition in an in vitro setting, and compared to five mM of D-glucose (normal glucose, or LG). The effect of co-culturing these cells with an ideal blood serum concentration of cholecalciferol-D3 and tocopherol was also examined. The current study aimed to examine the role of hyperglycemia in pulmonary arterial hypertension by the quantification and detection of the metabolomic alteration of smooth muscle cells in high-glucose conditions. Untargeted metabolomics was carried out using hydrophilic interaction liquid chromatography and high-resolution mass spectrometry. Cell proliferation was assessed by cell viability and the [^3^H] thymidine incorporation assay, and the redox state within the cells was examined by measuring reactive oxygen species (ROS) generation. The results demonstrated that PASMCs in high glucose (HG) grew, proliferated faster, and generated higher levels of superoxide anion (O_2_·^−^) and hydrogen peroxide (H_2_O_2_). The metabolomics of cells cultured in HG showed that the carbohydrate pathway, especially that of the upper glycolytic pathway metabolites, was influenced by the activation of the oxidation pathway: the pentose phosphate pathway (PPP). The amount of amino acids such as aspartate and glutathione reduced via HG, while glutathione disulfide, N6-Acetyl-L-lysine, glutamate, and 5-aminopentanoate increased. Lipids either as fatty acids or glycerophospholipids were downregulated in most of the metabolites, with the exception of docosatetraenoic acid and PG (16:0/16:1(9Z)). Purine and pyrimidine were influenced by hyperglycaemia following PPP oxidation. The results in addition showed that cells exposed to 25 mM of glucose were oxidatively stressed comparing to those cultured in five mM of glucose. Cholecalciferol (D3, or vitamin D) and tocopherol (vitamin E) were shown to restore the redox status of many metabolic pathways.

## 1. Introduction

Pulmonary artery hypertension (PAH) is a disease characterized by an increase in mean pulmonary arterial pressure by more than 25 mmHg, as well as vasoconstriction and vascular obstruction. This in turn increases pulmonary vascular resistance and right ventricular failure. Various factors contribute to pulmonary hypertension, so it is difficult to understand the mechanisms that initiate and develop the disease. Although knowledge of disease pathogenesis and treatment has greatly improved, the mortality rate is still high among PAH patients [1], and the survival of patients is significantly influenced, despite the classification of the disease [2,3]. The clinical diagnosis and treatment of PAH depends on the disease classification based on the “updated clinical classification of pulmonary hypertension”, while a comorbid condition such as hyperglycemia has not been taken into consideration yet [4]. The United Kingdom (UK) and Ireland pulmonary hypertension registry data shows that more than 20% of pulmonary hypertension (PH) patients above 50 years old had diabetes [5]. These data are associated with the combined data of four pulmonary hypertension centers that assessed diabetes mellitus for those who were newly diagnosed with PH, and found that 107 out of 415 pulmonary hypertension patients had diabetes [6,7]. On top of that, mortality was found to be higher among diabetic pulmonary hypertension patients compared to non-diabetic patients [6], indicating that hyperglycemia could adversely contribute to the PAH patients’ survival. Another study that investigated the survival of PAH patients with and without diabetes mellitus (DM) found that DM patients had 10 years lower survival than PAH patients without DM [7].

Based on that and other studies [8,9] that found glucose intolerance, insulin resistance, and metabolic syndrome influenced PAH, it can be hypothesized that hyperglycemia could disturb the metabolic pathway of PASMCs, leading to medial hypertrophy, fibrosis, proliferation, vessel thickness, and vascular inflammation and remodeling, leading eventually to right ventricular failure.

The study aimed to examine the alteration of the metabolomic profile that occurs in the pulmonary artery smooth muscle cells following an increase in glucose concentration, and the effect of antioxidant treatments such as cholecalciferol (D3, or vitamin D) or tocopherol (vitamin E) on the metabolomics of PASMCs during diabetic-likes conditions.

Evidence indicates that high glucose levels stimulate the overproduction of the reactive oxygen species (ROS) of vascular cells, which is considered to be strong contributor to the oxidative stress involved in the pathogenesis of subsequent complications in cardiovascular diseases [10]. Sustained exposure to elevated glucose levels enhances the production of reactive species in the heart and macrovascular system, and stimulates in turn fatty acid oxidation [10]. The overproduction of ROS by the mitochondria due to more glucose being oxidized in the trichloroacetic acid (TCA) cycle drives the mechanisms that are responsible for tissue damage [11]. Superoxide (O_2_·^−^) is the primary form of the mitochondrial free radical that transforms to other reactive species, leading to cell damage [12].

The overproduction of reactive species ultimately influences living organisms, as they interfere with cell DNA, lipids, and proteins, leading to mutation due to negative gene–environment interactions [13]. Cardiovascular diseases, diabetes, and neurodegeneration are the most common consequences of increased DNA oxidation. Increased lipid and protein oxidative damage in diabetes patients are associated with disease complications [14,15].

Glutathione (GSH) is an important and abundant endogenous that preserves the redox state of the thiol groups (R-SH) of proteins, which are essential for the restoration and expression of DNA. It serves as a scavenger of OH· and superoxide, detoxifies H_2_O_2_ and lipid peroxides, and supports the recycling of vitamins C and E [16]. A decrease in the cellular concentration of reduced GSH and glutathione metabolism impairment as a result of a reduction in cysteine and glycine, which are the precursors necessary to synthesize glutathione, has been correlated with diabetes [17,18]. Chronic hyperglycemia produces oxidative stress via the competition between glutathione reductase and aldose reductase for nicotinamide adenine dinucleotide phosphate reduced form NADPH, resulting in decreased GSH restoration from its oxidized form and a decreased ratio of NADPH/NADP (Oxidized form of nicotinamide adenine dinucleotide phosphate) [19].

**Figure 1:**
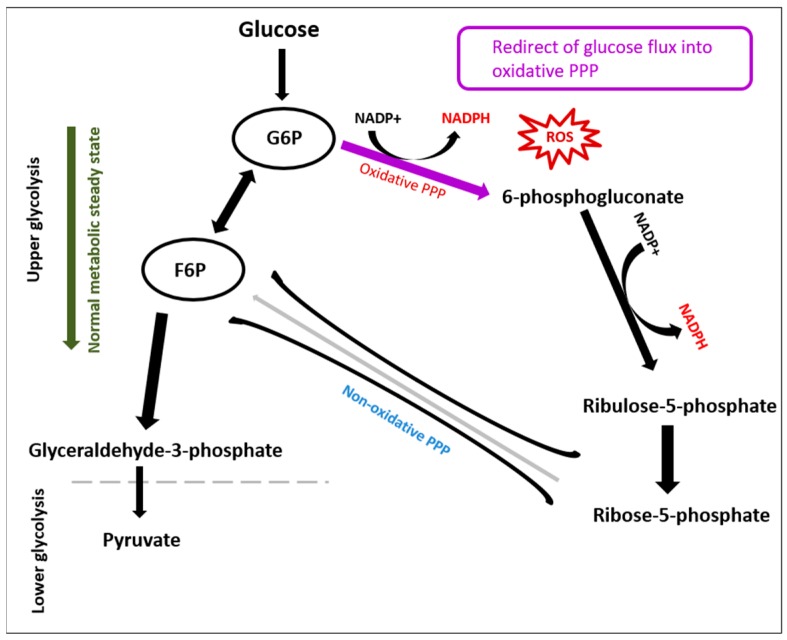
The rerouting of glucose flux into the pentose phosphate pathway (PPP) due to oxidative stress. Glycolysis was encouraged, while the activation of PPP by reactive oxygen species enhances the production of NADPH from NADP+.

**Figure 2:**
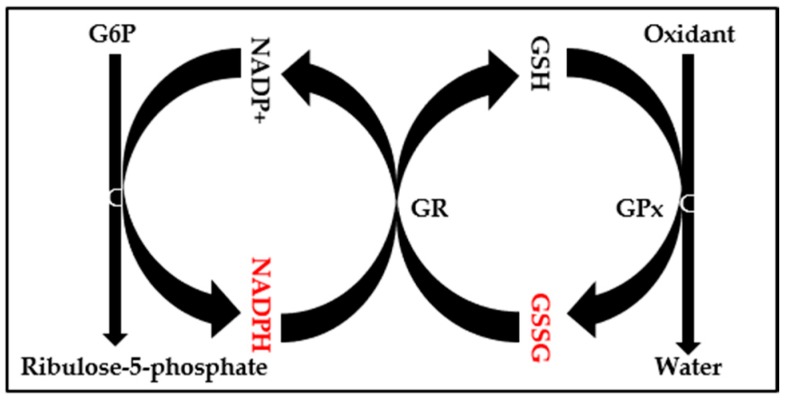
The oxidative branch of the PPP is a major supplier of NADPH for glutathione recycling. The reduction of glutathione (GSH) disulfide to glutathione via glutathione reductase needs NADPH to be completed. Thus, oxidized glutathione (GSSG):GSH and NADPH:NADP+ ratios are impaired due to oxidative stress.

ROS also obstructs glycolytic enzymes such as glyceraldehyde 3-phosphate dehydrogenase, phosphofructokinase-1, and pyruvate kinase M2. Proportionally, glycolytic inhibition diverts glycolysis toward the pentose phosphate pathway (PPP) oxidative arm to generate NADPH, which recycles oxidized glutathione as an antioxidant response to the oxidative stress (Figure 1 and Figure 2).

A decrease in the GSH/oxidized glutathione (GSSG) ratio has been reported as an oxidative stress marker. A study that observed the serum glutathione levels of diabetes patients found lower levels of GSH compared to control subjects [20].

ROS in addition can damage proteins, causing a loss of protein catalytic activity, and driving them to become more susceptible to proteolytic degradation [21].

Lipids also are vulnerable targets of ROS, as their molecular structures have abundant reactive double bonds [22]. Reactive species cause lipid peroxidation and disrupt the lipid bilayer, and this may deactivate membrane-bound receptors and enzymes, leading to a loss of membrane fluidity, elasticity, permeability, impaired cellular function, and cell lysis. Lipid peroxidation leads to products such as malondialdehyde (MDA), isoprostanes, and 4-hydroxynonenal. These have been identified as significant oxidative stress biomarkers, and are able to deactivate various cellular proteins by the formation of protein cross-links.

MDA is a reactive carbonyl compound that is known to be mutagenic, atherogenic, and carcinogenic. Its reaction with lysine residues forms lysine–lysine cross-links, which have been detected in apolipoprotein B (apoB) fragments of oxidized low-density lipoprotein (oxLDL), and has been hypothesized to interrupt the interaction between oxLDL and macrophages, and thereby enhance atherosclerosis [23].

### Antioxidants

Vitamins D and E have been shown to have antioxidant properties [24]. The serum levels of 25-hydroxy vitamin D are found to be deficient among type 2 diabetic patients [24,25]. Animal models and clinical trial studies have demonstrated the ability of vitamin D to reduce oxidative markers [26,27]. Vitamin E is a lipid-soluble antioxidant that scavenges lipid radicals. It has been reported that the incidence of primary major coronary events among subjects who received vitamin E decreased by 4% compared to a control group receiving placebo, and the incidence of primary major coronary events decreased by 8% [28]. However, other trial studies indicated that the efficacy of vitamin E in reducing the number of major cardiovascular incidents did not influence total mortality [29,30]. The current study aimed to investigate the role of both vitamin E and D as antioxidants, and examine their effects on the smooth muscle cell metabolome in the presence of high-glucose media.

## 2. Results

### 2.1. Effect of High-Glucose Media Alone, with Vitamin D, or with Vitamin E, on Cell Viability

**Figure 3:**
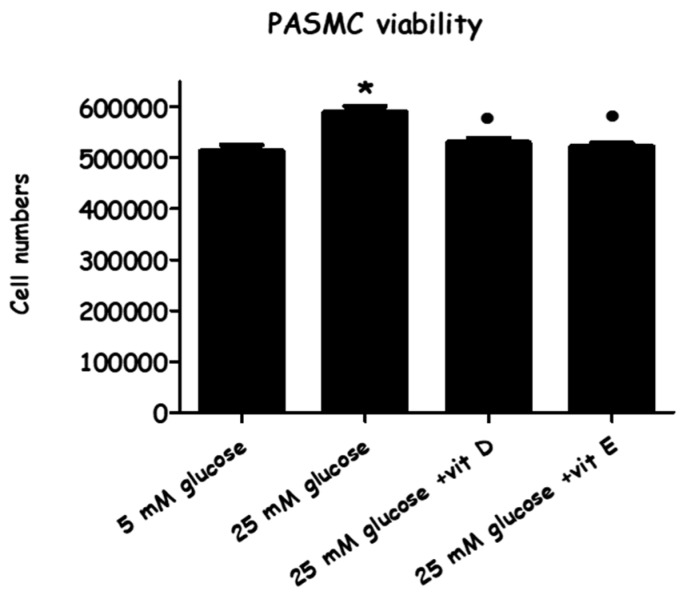
The effect of vitamin D/vitamin E in high glucose-induced pulmonary artery smooth muscle cells (PASMC) proliferation. Cells were cultured with normal glucose (five mM) and high glucose (25 mM) for 72 h. Vitamin D (80 ng/mL) and Vitamin E (9 µg/mL) were added immediately along with the high-glucose incubation. Cells were stained with trypan blue dye, and the viable cells were counted after 72 h. Each value represents the mean ± SEM from four independent experiments. * *p* < 0.05 vs. cells cultured in normal glucose (five mM) media. ● *p* < 0.05 vs. high glucose stimulated cells. ANOVA one way followed by Tukey’s comparison test was performed.

Prior to cell extraction preparation for LC/MS, the high and normal glucose cultured cells and the treated cells were counted using trypan blue exclusion at the end of the 72-h experimental period (Figure 3). This demonstrated that viable cell numbers were substantially increased by the high-glucose (HG) culture medium (14.9 ± 0.67% versus normal glucose, or LG). Treating pulmonary artery smooth muscle cells (PASMCs) with vitamin E or vitamin D decreased cell numbers almost to the level of the normal glucose medium (by 11.5 ± 0.4% or by 10.1 ± 2.3% versus HG), respectively.

### 2.2. Effect of High-Glucose Media Alone, with Vitamin D, or with Vitamin E on PASMCs’ Proliferation

**Figure 4:**
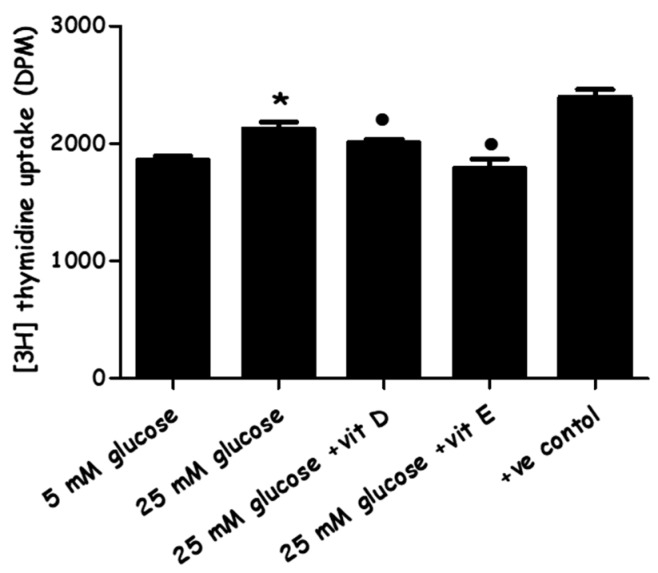
The effect of vitamin D and vitamin E on high glucose-induced [^3^H] thymidine uptake by PASMCs. Quiesced cells were cultured with normal (five mM) and high glucose (25 mM) for 72 h. Vitamin D (80 ng/mL) and vitamin E (9 µg/mL) were added immediately along with high-glucose incubation. [^3^H]-thymidine incorporation assay was performed for the evaluation of DNA synthesis (as an index of cell proliferation). Radioactive counts were measured in disintegrations per minutes (DPMs) ± SEM. * *p* < 0.05 vs. cells cultured in normal glucose (five mM) media. ● *p* < 0.05 vs. high glucose cultured cells (*n* = 4). ANOVA one way followed by Tukey’s comparison test were performed. (+ve) control: cells placed in culture of ordinary environment with no adjusted glucose and 10% bovine serum.

The proliferative capacity of high-glucose media and the combined effects of high glucose with vitamin D/or with vitamin E on cell proliferation were assessed by the [^3^H] thymidine incorporation assay. High-glucose media increased DNA synthesis in PASMCs by (14.4 ± 3.19% versus LG), while vitamin D and vitamin E were significantly able to suppress the DNA synthesis of cells cultured in high glucose by (5.3 ± 2.8% and 15.6 ± 4%, versus HG), respectively (Figure 4). Cell counting and proliferation quantification showed that for cells cultured at matching density and examined (counting or assayed) at the same time point of 72 h, the PASMCs in the HG media steadily revealed a noticeably superior proliferation rate.

### 2.3. Evaluation of Oxidative Stress

The measurement of ROS was carried out to determine whether or not the high concentration of glucose in the cultured media enhanced oxidative stress activity within the cells. In addition, ROS levels were also evaluated following the combined effects of HG media supplemented with vitamin D/and with vitamin E.

**Figure 5:**
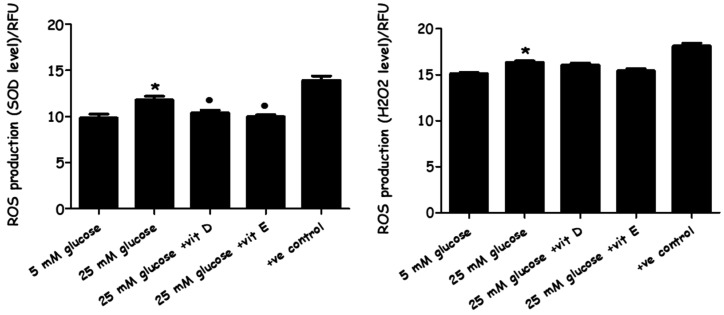
The effect of high-glucose media alone, with vitamin D, and with vitamin E on reactive oxygen species (ROS) production. PASMC cells were incubated with normal (five mM) and high glucose (25 mM) for 72 h. Vitamin D (80 ng/mL) and vitamin E (nine µg/mL) were added immediately along with high-glucose incubation. Superoxide (O_2_·^−^) levels and hydrogen peroxide (H_2_O_2_) levels were detected by dihydroethidium (DHE) and 2′, 7′-dichlorofluorescein (DCF) fluorescence compound, respectively. * *p* < 0.05 vs. unstimulated cells (*n* = 4). * *p* < 0.05 vs. cells cultured in normal glucose (five mM) media. ● *p* < 0.05 vs. high-glucose cultured cells *n* = 4). ANOVA one-way followed by Tukey’s comparison test was performed. (+ve) control: cells were treated by ROS inducer agents.

Intracellular superoxide (O_2_·^−^) levels and hydrogen peroxide (H_2_O_2_) release within the PASMCs were measured by dihydroethidium (DHE) and 2′, 7′-dichlorofluorescein (DCF), respectively. The results showed that PASMCs cultured in HG media significantly generated higher ROS (superoxide and H_2_O_2_) levels in comparison to cells cultured in LG media. Interestingly, adding vitamin D or E to the high glucose-cultured cells significantly reduced the intracellular superoxide levels, while no effect was noticed on hydrogen peroxide (Figure 5). So, they were able to reduce the primary ROS product (O_2_·^−^), but not the secondary ROS product (H_2_O_2_). 

### 2.4. Metabolomics

LC/MS analysis was carried out where the samples were prepared and injected, and data afterwards was extracted and processed to identify all of the biochemical (metabolite) markers changed by HG media comparing to control (LG). Then, the impact of adding vitamin D and vitamin E on HG media were also measured on those altered metabolites. Triplicate samples were prepared for each group, and only consistent metabolites alterations were listed.

#### 2.4.1. Quality Control Samples (Pooled)

**Figure 6:**
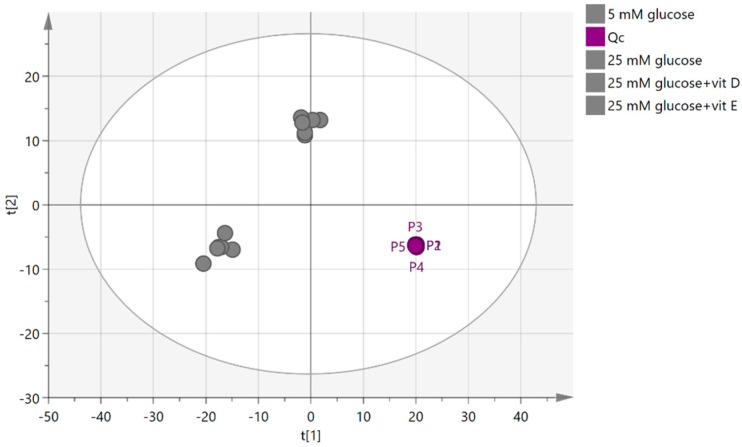
Principal components analysis (PCA) score plot for quality control (QC) (pooled) cell extract samples of (PASMCs). P1, P2, P3, P4, and P5 represent the quality control sample runs frequently throughout the experiment.

To measure the precision of the instrument, the relative standard deviation (RSD) was calculated based on the reading of a pooled sample, which was injected five times during the course of the run and clustered together in the principal components analysis (PCA) plot (Figure 6). An RSD was calculated based on the total intensities in each sample, and an RSD of 19.58% was obtained. RSD was then calculated individually for each putative metabolite in the pooled sample, and the highest RSD was for oxoglutarate (138%) followed by ornaline (91%), while glutamine had the lowest RSD value (0.076%). The precision of these values clearly indicates that any metabolic differences between the groups cannot be due to instrumental factors alone. Metabolites with RSD values higher than 20% were excluded before the model was fitted by using Umetrics multivariate tool multivariant analysis application (SIMCA-P).

#### 2.4.2. Visualization and Biomarkers Identification

Principle component analysis (PCA) was employed to observe the possible presence of outliers, groups, similarities, and other patterns in the data. Afterward, Orthogonal Projections to Latent Structures Discriminant Analysis (OPLS-DA) was used to understand the differences between groups and identify the biomarkers that distinguish the groups from one another.

**Figure 7:**
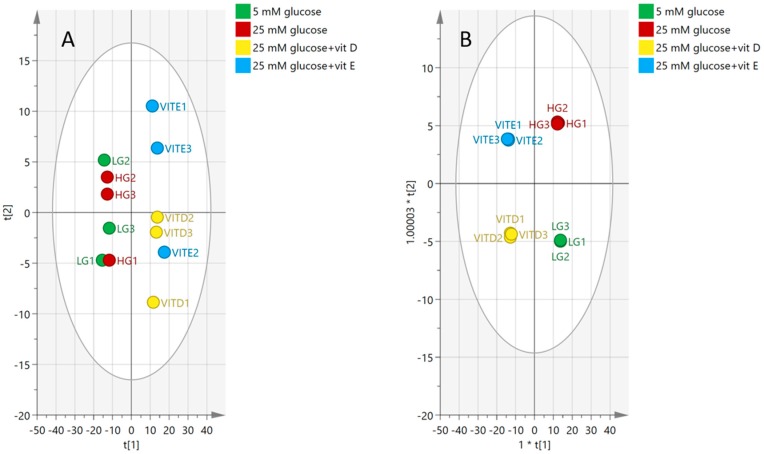
(**A**) Principal components analysis (PCA) plot, (**B**) Orthogonal Projections to Latent Structures Discriminant Analysis (OPLS-DA) plot for groups of normal glucose (five mM glucose), high glucose (25 mM glucose), vitamin D, and vitamin E. The plot shows the distribution of 12 samples based on the reading of 549 putative metabolites.

The PCA plot in Figure 7 shows the separation between groups of cells cultured in vitamins D and E, and another two groups cultured in high and normal glucose alone. No clear separation can be seen between the normal (LG) and high (HG) glucose groups (Figure 7A). However, clustering became clearer when OPLS-DA supervised analysis was applied.

The OPLS-DA model shows a clear clustering pattern in which each group was able to gather, as can be seen in Figure 7B. Even though samples were clustered, goodness of prediction was high at 0.99, and the model fit was good (72% predictive, 23% orthogonal), the cross-validated (CV)-ANOVA of the model (*p* = 0.885) indicated that the model was not valid (Table 1).

#### 2.4.3. Effect of High-Glucose Media

In order to identify the markers that distinguish the HG and LG groups, a new model was built comparing only samples cultured in LG and HG. The, the data were filtered based on CV-ANOVA, with 95% confidence intervals and variables of importance for the projection (VIP) values of each of 549 features, as described in Figure S1. This workflow highlighted 80 metabolites that distinguished the HG group from the LG group. *p* values < 0.05, 95% CI not including zero values, RSD <20% in the pooled samples, and VIP values >1.

**Figure 8:**
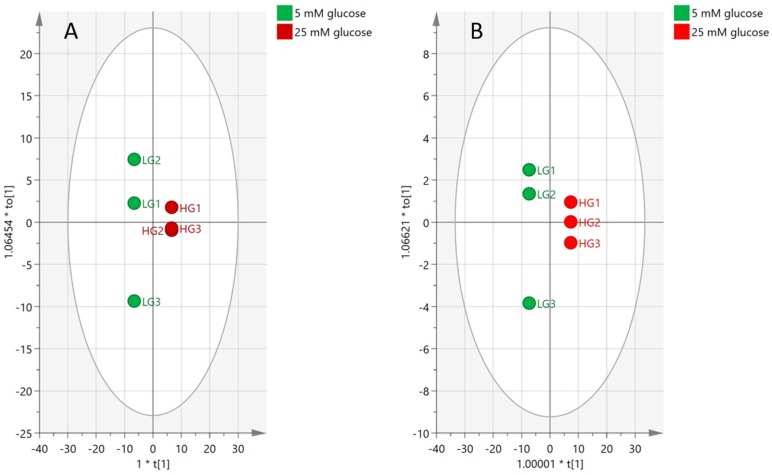
OPLS-DA score plots showing the effect of high-glucose media compared to normal glucose media on the pulmonary artery smooth muscle cells. (**A**) Based on 549 features. (**B**) Based on 80 significant markers affected by hyperglycemic media.

Figure 8A shows the samples clustering in the model based on 549 metabolites, while the model fit 89% of the data (30% predictive, 59% orthogonal and CV-ANOVA, *p* = 0.0954). Plot (B) was then rebuilt based on the reading of 80 significant biomarkers where the model was able to fit 86% of the data (80% predictive, 6% orthogonal, and CV-ANOVA, *p* = 0.0141).

Several lipids and lysolipids fell significantly due to high glucose, indicating that the cell membrane composition was affected. Some amino acids were influenced too, especially lysine degradation and biosynthesis, while in addition, oxidized glutathione (GSSG) as an oxidative damage marker increased, and glutathione (GSH) decreased. Glycolysis metabolites were influenced as well when particularly the pentose phosphate oxidative pathway was activated due to high-glucose flux into that pathway. In addition, NADH was slightly increased, while Acetyl-coA and TCA acids were not influenced, suggesting that lower glycolysis was not as active as that at the upper site. ATP was decreased, which may indicate that it was consumed in cell division, while the cell growth increased when PASMCs were cultured in HG media. In addition, the majority of the nucleotides’ metabolites were influenced by HG following the activation of pentose phosphate pathway (PPP).

### 2.5. Antioxidants

Metabolic profiling illustrated that using vitamins D and E attenuated the oxidative stress that occurred through exposing PASMCs to HG.

Many of the metabolites see (Supplementary Materials, Table S1) that were altered by HG were restored to control levels when plasma concentrations of either vitamin D or E were added. Glycolysis metabolites that were enhanced by HG illustrated showed a contrary response when the antioxidants were used. This effect also can be seen in oxidized and reduced glutathione, where particularly vitamin D helps to preserve normal status. Many lipids, including fatty acids and glycerophospholipids (GPL), were decreased by HG, but the antioxidants were not specifically effective with GPL to restore most of the altered metabolites. Nucleotide metabolites generally increased due to HG, while antioxidants were able to restore most of them to around normal levels.

## 3. Discussion

High glucose (HG) is an important cause of cardiovascular disease, which acts via various mechanisms to enhance ROS accumulation. The accumulation of ROS successively causes cellular impairment, which is strongly involved in diabetes complications. ROS can immediately damage cellular components, including proteins, carbohydrates, lipids, DNA, and subsequently affect the metabolic pathways, resulting in a shift of (PASMC) phenotype from the contractile phenotype to a synthetic (hyperproliferative/apoptotic-resistant) phenotype, and initiating cell proliferation and migration [31]. While, in turn, some clinical studies have found inadequate provision for the use of antioxidants in diabetes [32,33,34], others have demonstrated the advantages of antioxidant usage in the prevention of diabetic cardiovascular complications [35,36,37].

The current study was designed to determine the impact of HG on PASMC proliferation and ROS generation. It focused on the abnormal changes in the cellular metabolome in order to investigate the effect of adding the antioxidants vitamin D or E on the prevention of cell proliferation and the oxidative damage caused by ROS generation, which could be induced by stimulating the PASMCs with HG medium.

The data obtained from the cell viability assay demonstrated a significant increase in the number of PASMCs by increasing the glucose concentration to 25 mM (Figure 3). This result is compatible with the results of the [^3^H] thymidine incorporation assay (Figure 4), which indicated that cell proliferation increased in response to HG, as an increase in both total cell numbers and DNA synthesis were observed. Cell proliferation was slightly but statistically significantly induced when cells were exposed to HG for 72 hours while, in turn, adding vitamin D or E to HG media succeeded in inhibiting cell proliferation. These results link with many studies carried out on animal and human cellular models [38,39,40], indicating that HG medium enhances pulmonary arterial smooth muscle cell proliferation via different pathways. In contrast, the antioxidants, particularly vitamins D and E, have been found to suppress vascular smooth muscle cells VSMCs’ proliferative activity [41,42,43] through different mechanisms.

The current results also showed that HG medium not only induced cell proliferation, but also elevated ROS levels, including both superoxide and H_2_O_2_ levels (Figure 5). Vitamin D inhibited the overexpression of superoxide O₂⁻ in high-glucose stimulated cells, but not H_2_O_2_, while vitamin E was able to suppress both O_2_^−^ and H_2_O_2_ levels. Vitamin D is well-known to promote the scavenging of ROS and diminish reactive species formation in diabetic mice by the suppression of NADPH oxidase gene expression [44,45]. In an experiment carried out on diabetic mice, vitamins D and E decreased lipid peroxidation and promoted superoxide dismutase (SOD) activity [46]. Alpha-tocopherol (vitamin E) inhibited the prevalence of peroxidation originating from reactive species, preventing undesired lipid oxidation [47].

**Figure 9:**
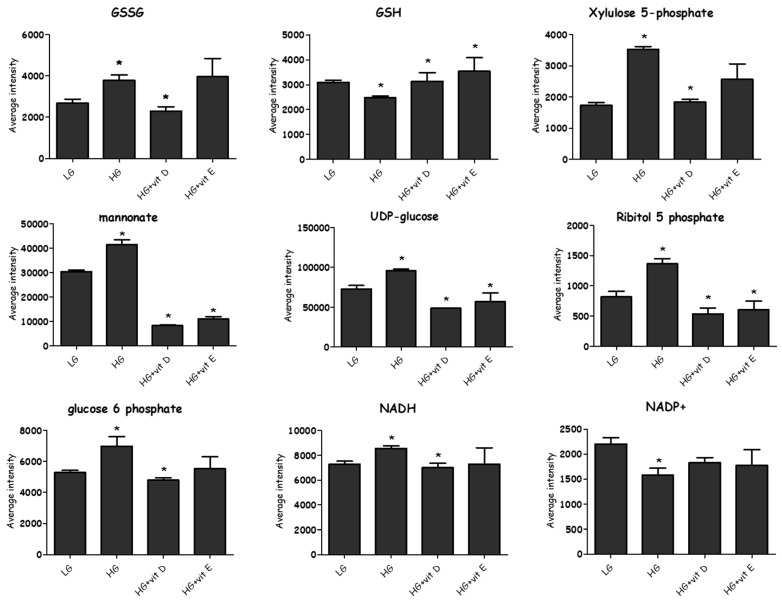
Changes occurred among selected metabolites due to different interventions. Most of the glutathione hemostasis and PPP metabolites influenced by hyperglycemia responded to antioxidants and preserved the normal levels. Pentose phosphate specifically was attenuated via vitamins D and E, suggesting that NADPH re-equipment was decreased. (*) (CV-ANOVA < 0.05).

In the current experiment, adding physiological concentrations of vitamin E and vitamin D to the stimulated cells had a favorable effect on most of the altered metabolites. A total of 549 metabolites were profiled in both positive and negative modes by LC-MS, while only 80 metabolites were found to distinguish HG samples from LG in a valid OPLS-DA model. Samples treated with vitamin E or vitamin D were also compared to the HG group to examine their impact as antioxidants. The impact of the antioxidants on the 80 metabolites that distinguished the LG from HG was only considered. Vitamin E and vitamin D restored many of the metabolites affected by HG to the levels observed in LG (Figure 9).

### 3.1. Glycolysis

**Figure 10:**
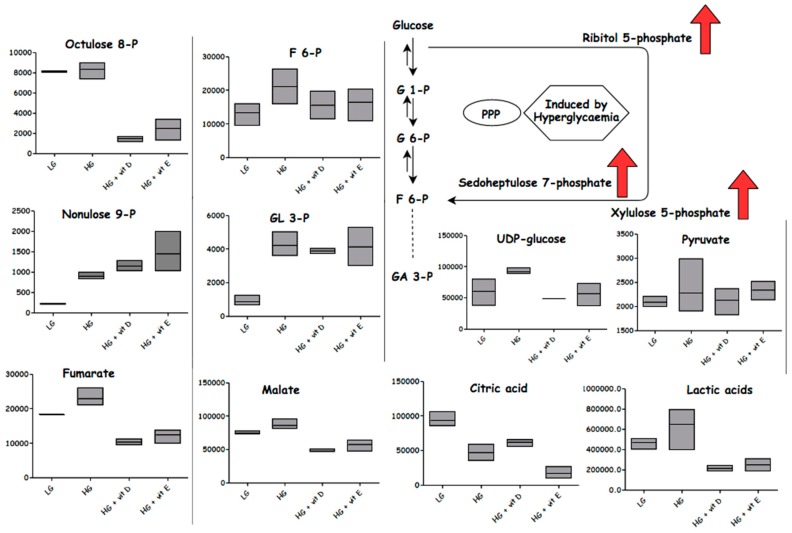
Metabolites affected by different conditions: normoglycemia (LG), hyperglycemia (HG), with vitamin D or vitamin E. Column figures display the average intensities of specific metabolites in different conditions. G1-P: glucose 6-phosphate; G6-P: glucose 6-phosphate; GA 3-P: 3-phosphoglyceraldehyde; F6-P: fructose 6-phosphate.

Glucose metabolism fluctuation plays an important role increasing cellular oxidative stress and the vascular remodeling of PAH in diabetic patients [48,49]. In the current study, although increased levels of glucose-1-phosphate (G-1P) and glucose-6-phosphate (G-6P) were observed, as well as increased levels of glyceraldehyde phosphate and dihydroxy acetone phosphate in HG compared to LG, vitamin D and vitamin E had so significant effect in lowering these metabolites. HG samples revealed higher levels of gluconate, cytidine diphosphate (CDP)-ribitol, ribitol 5-phosphate, uridine diphosphate (UDP)-glucose, and xylulose 5-phosphate, indicating that the glucose flux was redirected toward the PPP. The shift of glucose flux toward the PPP is compatible with increased cell proliferation, reflecting an increased requirement for nucleotides. Stimulated smooth muscle cells activate PPP in order to generate NADPH from NADP+ as part of an antioxidant mechanism. However, it would seem that the flux through the PPP is via a non-oxidative route into sedoheptulose phosphate, since the levels of NADPH are reduced possibly as a result of excessive ROS generation, as evidenced by an increase in GSSG levels [50]. The elevation of the non-oxidative route into sedoheptulose phosphate is underlined by the elevation of compounds putatively identified as octulose and nonulose phosphates, which could be formed via the action of transaldolase on fructose, ribose phosphates, and two fructose phosphate molecules, respectively. Interestingly, phosphoglycerate was not increased by HG, which might suggest further reinforcing the view that there was a diversion of glucose into the PPP upstream of these metabolites. HG samples exhibited a slight increase in their NADH levels, which indicated that the Krebs cycle was not significantly inhibited, and suggested that other pathways such as the polyol pathway may be induced by hyperglycemia see (Figure 10). The TCA cycle acids mainly were not increased by HG, with a decrease of citric acid and increase of fumaric acid. Lactate in addition was slightly increased, which could also indicate that there was some conversion of pyruvate to lactate, rather than the acetyl coenzyme A (acetyl coA) “Warburg effect”. The accumulation of 3-phosphoglyceraldehyde (GA 3-P) was significantly increased in the HG samples, indicating that excessive ROS generation might inhibit the conversion of most of the GA 3-P via the glycolysis process into pyruvate. N2-(D-1-carboxyethyl)-L-lysine (CL) is a product of the reaction of methylglyoxal (MG) with lysine residues in proteins, which occurs in diabetes, and has been proposed to be linked to cardiovascular disease and indicates increased levels of MG. MG has been found to inhibit glyceraldehyde phosphate dehydrogenase, thus causing the accumulation of GA 3-P and the diversion of GA 3-P into the PPP [51]. Reaction with NADP+ and H_2_O in the presence of CL dehydrogenase forms L-lysine, NADPH, and pyruvate. In the HG samples, the accumulation of CL pointed to the inhibition of that reaction. Vitamin D and vitamin E appear to have a direct effect in promoting the degradation of CL. Antioxidants showed no impact on the GA 3-P that was upregulated by HG media. These findings confirm that the upper glycolysis and PPP were strongly affected by HG, but the lower glycolysis and TCA cycle were not.

**Figure 11:**
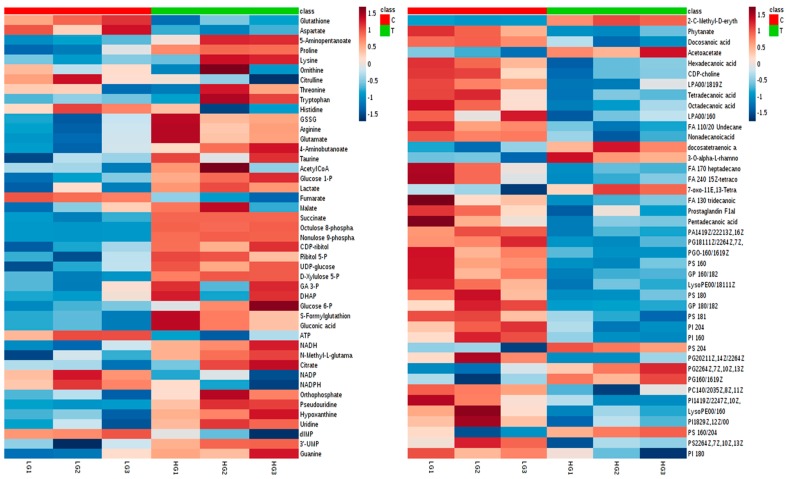
Heat map visualizing the significant metabolites impacted by high glucose (HG) comparing to normal glucose (LG) conditions. Row: represents the metabolite; Column: represents the samples group (red: normal glucose; green: high glucose); The color key specifies the metabolite intensity; lowest: dark blue; highest: dark red.

Under HG, the cells appear to produce glycogens with UDP glucose (their precursor) and sugar dimers, tetramers, and pentamers being increased, indicating that the cells have the capacity for glucose storage. ATP levels were lowered by HG, indicating that increased energy was consumed, which may correlate with increased cell proliferation. These findings agree with different studies that have indicated that high glucose produces oxidative stress, disturbed glucose metabolism, and impaired the most substantial source of energy—“electron transport chain” (ETC)—activity, causing ETC dysfunction [51,52,53,54]. Vitamin D or E attenuate many of the metabolites induced by HG; see (Figure 11). Vitamin D decreased the level of GSSG and increased the level of GSH; this was mirrored by a further fall in NADPH in comparison with HG, since it is consumed as a co-factor in the conversion of GSSG to GSH. Correspondingly, GSH levels increased in the HG samples treated with vitamin D. GSH levels also increased in the vitamin E-treated samples, but without GSH or NADPH falling, suggesting a different mechanism for conserving GSH compared with vitamin D. HG is believed to diminish GSH synthesis, which is reported in diabetic patients [55]. Increasing GSSG formation is associated with the excessive generation of free radicals as a marker of oxidative stress, which are believed to be elevated in diabetes and PAH [55,56]. Despite the decrease in ATP when antioxidants were added, NADH and NADP+ were restored almost to their normal levels. 

### 3.2. Amino Acids

Amino acids were generally disturbed with no clear pattern, while aspartate downregulation may indicate an inhibition of the conversion of aspartate to pyruvate, which has been demonstrated previously in a metabolomics-based study of pulmonary arterial hypertension [57]. Citrulline and histidine were also downregulated, while almost all of the other amino acids were not significantly affected, apart from glutamate and N2-(D-1-Carboxyethyl)-L-lysine, which were increased by HG. In addition, N6-acetyllysine and aminopentanoate were influenced, indicating that the lysine degradation pathway might be activated in order to promote the biosynthesis of acetoacetyl-CoA. Although small changes of amino acids appeared due to HG, vitamins D and E were able to inhibit some of the induced changes such as those for glutamate, N2-(D-1-Carboxyethyl)-L-lysine, and aminopentanoate.

### 3.3. Lipids

The metabolic profiling of induced PASMCs showed an alteration of lipid pathways by HG. However, there was no clear pattern, with some lipids, especially fatty acids being upregulated and some being downregulated by HG. The clearest effect of vitamin D and vitamin E appeared to be in promoting increased levels of the polyunsaturated fatty acids linoleic acid, eicosatrienoic acid, docosatetraenoic acid, and docosatrienoic acid. This might indicate that some of these acids protect against oxidation. A study carried out on the lipid metabolism of vascular cell physiology showed that cardiovascular diseases were associated with changes in lipid metabolism, and accordingly alterations in lipid profiles [58]. Other studies carried out on Alzheimer’s disease revealed a significant decrease in long-chain glycerophosphocholine (PC) (36:5; 38:6 and 40:6), sulfatides [59,60], and increases of very long-chain lysophosphatidylcholine (LPCs) (32:0 and 34:0) [52]. Vitamin E is an effective antioxidant reserve lipid, avoiding most lipid oxidation [53]. Vitamin D was firstly identified to work as antioxidant in 1993, when it was shown that it had the ability to prevent lipid peroxidation in the cell membrane [24]. However, our result demonstrated a narrow impact on specific lipids that could show a positive response to the antioxidants, suggesting that they might of help to prevent little of the undesired modifications occurring on the lipid metabolites due to HG.

### 3.4. Nucleotides

HG induced pyrimidine and purine pathway metabolites and increased the intracellular content of uridine, pseudouridine, 3′-uridinemonophosphate (UMP), guanine, and hypoxanthine. PPP influences nucleotide metabolites that are believed to modulate smooth muscle cell proliferation. Cell culture studies on artery SMCs derived from human and rat showed that mononucleotide polyphosphates such as ATP and uridine-triphosphate (UTP) stimulate SMC proliferation [61]. In vitro and in vivo studies found that increases of the intracellular content of uridine correlated with an increase in insulin resistance [62,63]. Vitamins D and E have a marked effect on lowering uridine levels under HG. A study that investigated biochemical oxidative markers found that hypoxanthine plasma concentration was elevated [54] among patients of obstructive sleep apnoea syndrome, which is a disease characterized by an increase of oxidative stress in comparison to normal subjects. However, hypoxanthine has also been found to inhibit the proliferation of smooth muscle cells, and both antioxidant vitamins were found to greatly increase hypoxanthine levels in the current experiment.

## 4. Materials and Methods

### 4.1. Cell Culture

Rat pulmonary artery smooth muscle cells (PASMCs) were obtained, harvested, and dissected from Sprague–Dawley rats and cleaned independently under aseptic conditions. Small rings of the pulmonary arteries were transferred into 25-cm^2^ flasks that were filled with culture media. Cells were then supplemented by 10% bovine calf serum (BCS), 5% penicillin, and streptomycin in mixture of 1:1 F-12: Dulbecco’s Modified Eagle’s medium (DMEM) and cultured at 37 °C in a 21% O_2_, 5% CO_2_ incubator. In addition, the condition of each group was prepared as follows: (1) 25 mM of glucose for high-glucose media “diabetic-like condition”; (2) five mM of glucose for normal glucose media (3) 25 mM of glucose + physiological concentration of vitamin D cholecalciferol (80 ng/mL) or (4) 25 mM of glucose + physiological concentration of vitamin E α-tocopherol (9 µg/mL).

### 4.2. Cell Viability

PASMCs were seeded at an intensity of 14 × 10^4^ cells/well in 25-cm^2^ flasks supplemented with 10% BCS DMEM overnight and allowed to attach. Next, cells were washed with free media and cultured in 5% BCS for 72 h with appropriate glucose and antioxidant concentration based on our intervention and the condition of each group. By the end of the incubation period, cells were harvested, suspended, and counted using a haemocytometer cell counting chamber. Ten percent of trypan blue was used to detect viable cells.

### 4.3. PASMC Proliferation Assay

Confluent PASMC were trypsinized, placed in DMEM supplemented with 10% BCS, and seeded into 24-well flasks at a cell intensity of 2 × 10^4^ per well. After 24 h, cells were washed by free media and quiesced with 0.1% BCS for 24 h. Cells were then cultured in 5% BCS for 72 h with appropriate glucose concentrations and antioxidant concentrations based on our intervention and the condition of each group. During last six hours of the assay, [^3^H-thymidine] aqueous solution (GE Healthcare^®^) was added to each well. In order to prepare assay samples, media were discarded and cells were rinsed by one milliliter of cold PBS for 10 minutes and treated four times with one milliliter of 10% (*w*/*w*) trichloroacetic acid (TCA, Sigma). Afterward, 250 μL 0.1% sodium hydroxide/sodium lauryl sulphate (SDS) added into each well and two milliliters of Emulsifier-safe™ scintillation fluid was added to the scintillation vial of each sample before they were mixed well prior to measuring the radioactive counts using a scintillation counter.

### 4.4. Reactive Oxygen Species Experiment

PASMCs were seeded at 5 × 10^3^ cells per well in 96-well plates supplemented with 10% BCS DMEM for 24 h and allowed to attach. Then, the media was discarded, and the cells were washed by free media, followed by the addition of 5% BCS DMEM media for 72 h with appropriate glucose and antioxidant concentrations, based on our intervention and the condition of each group. During the final 30 minutes of the assay, 2.0 µL of dihydroethidium (DHE) or 10 µL of 2′, 7′-dichlorfluorescein (DCF) were added to each well, and the cells were placed for 30 minutes in the dark before fluorescence microscopy was performed. Dihydroethidium (DHE) and 10 µL of 2′, 7′-dichlorfluorescein (DCF) are fluorogenic dyes that measure reactive oxygen species (ROS) superoxide and hydrogen peroxide, respectively.

### 4.5. Metabolic Profiling Sample Preparation

In an appropriate number of replication, cells were cultured with various conditions at indicated final concentrations with their corresponding vehicle-only controls, and incubated for 72 h. Afterward, the medium was discarded, and the cells were twice washed with three mL of phosphate-buffered saline (PBS) at 37 °C before lysis. Volumes of the extraction solution were adjusted relying on the cell number of each condition, with one ml independently added to each 1 × 10^6^ cells. Cells’ flasks were then placed on ice where an appropriate volume of ice-cold extraction buffer (methanol:acetonitrile:water 50:30:20) was added, and cells were harvested using a cell scraper. Then, samples were vortexed for 12 minutes before they were placed into centrifuge at 0 °C for 15 minutes and 15,300 (r.p.m). Supernatant was collected and placed in −20 °C for further liquid chromatography mass spectrometer analysis (LC/MS). During the run, we maintained the temperature of the auto sampler at 4 °C. Mixtures of standard biochemicals (Sigma-Aldrich, Poole, UK) and the pooled quality control (QC) sample were injected throughout the analysis run in order to simplify the identification of metabolites and assess the method’s stability and reproducibility. The QC sample was prepared by taking equal aliquots from all of the samples and placing them into one HPLC vial.

### 4.6. Samples Analysis (LC/MS)

Analysis was carried out on an Accela HPLC system interfaced to an Exactive Orbitrap mass spectrometer (Thermo Fisher Scientific, Bremen, Germany) using a hydrophilic interaction liquid chromatography (HILIC) column (ZIC-pHILIC, 150 × 4.6 mm, 5 µm particle size) supplied by Hichrom Ltd. (Reading, UK). The system was initially purged by mobile phases followed by Zic-pHILIC column conditioning at an appropriate flow rate before the sample runs were carried out. Using standard lab method reported previously [64], samples were randomly placed in the autosampler tray. The ZIC-pHILIC mobile phase consisted of 20 mM of ammonium carbonate (Sigma-Aldrich, Poole, UK) in purified water at pH 9.2 (solvent A) and acetonitrile (Sigma-Aldrich, Poole, UK) (solvent B) at a flow rate of 0.3 mL/min. The elution gradient was an A:B ratio of 20:80 at 0 min, 80:20 at 30 min, 92:8 at 35 min, and finally, 20:80 at 45 min. The electro-spray ionization (ESI) interface worked in both positive and negative ion-switching modes, with +4.0 kV of spray voltage for the positive mode and −3.5 kV for the negative mode. The ion transfer capillary temperature was 320 °C, while the sheath and auxiliary gas were set at 51 and 17 arbitrary units, respectively. The full scan range was set at 75 *m*/*z* to 1200 *m*/*z*, with the automatic gain control (AGC) target and resolution as Balanced and High (1E6 and 50,000), respectively. Before analysis, mass calibration was done for both ESI modes via standard Thermo Calmix solution. The signals at 83.0604 *m/z* (2 × ACN + H) and 91.0037 *m*/*z* (2 × formate − H) were obtained as lock masses for both modes, respectively. Data were collected using the Xcalibur 2.1.0 software package (Thermo Fisher Scientific, Hemel Hempstead, UK).

### 4.7. Data Extraction and Analysis

Using MzMatch software (IDEOM) [65] with certain specifications was used to convert the Xcalibur peaks (see Figures S3 and S4) into numeric values, after which they could be processed and analyzed. Metabolites were detected to the metabolomics standards initiative (MSI) levels 1 or 2, which were primarily either based on exact mass (<three ppm deviation) plus retention time matching to a standard, or according to accurate mass. Data were then analyzed using univariate (Excel) and multivariate (SIMCA-P) to determine the most significant metabolites and pathways affected by intervention. Log transformation was applied on the data prior to the analysis and filtered finally on specific criteria (see Figures S1 and S2).

## 5. Conclusions

Sustained exposure to hyperglycemia causes the metabolic disruption of pulmonary artery smooth muscle. It can also lead to cellular damage, and could contribute to pulmonary hypertension via enhancing the generation of free radicals and promoting the proliferation of PASMCs, which eventually resulted in the narrowing and remodeling of the pulmonary arteries and pulmonary hypertension. Therapeutic stabilization and the restoration targeting of pulmonary arterial smooth muscle cell function via antioxidants treatment can demonstrate benefits for improving the redox state related to diabetic and cardiovascular deterioration. Based on that, consistently with the importance of avoiding ROS emerging by hyperglycemia to develop cardiovascular disease complication, antioxidants may assist in reducing the oxidative damage straight via reacting with free radicals by scavenging them, or indirectly by suppressing the free radical-generating enzymes or promoting intracellular antioxidant enzymes activity, which all could slow the proliferation of PASMCs. Vitamins D and E are antioxidants that are considered to maintain the redox state of PASMCs in the existence of an elevated glucose condition in an in vitro setting.

### Limitations and Future Prospects

Our findings were directly linked to cell-based work that is focused on the medial smooth muscle cell, which may not be particularly applicable in an in vivo setting. Inflammatory processes and immune system impacts were not taken into account. Thus, the reported responses in our hyperglycemic (diabetic-like) cell culture model might differ from the hyperglycemic responses in an in vivo setting. Therefore, it is a valuable to investigate the current study observations on an animal model. In addition, a further increase of the glucose levels and concentrations of vitamins might be considered in future studies.

## Figures and Tables

**Table 1:** The validity of the model shown in Figure 7B, where the P value was above 0.05.

Model	SS	DF	MS	F	*p* Value	SD
Total corr.	33	33	1	-	-	1
Regression	27.15	30	0.905	0.465028	0.885	0.9514
Residual	5.840	3	1.946	-	-	1.3952

## Supplementary Materials

The following are available online at http://www.mdpi.com/2218-1989/8/4/87/s1, Table S1: Significantly changed metabolites in PASMCs resulting from treatment with HG or HG + vitamin D or E. Figure S1: Data filtration steps. Figure S2: log2 transformation of the data improves data distribution. Figure S3: Extracted ion traces for GSSG in HG and LG media. Figure S4: Extracted ion traces for maltotetraose and maltopentaose in HG and LG media.
