# Supplementary material for: Untargeted Metabolic Profiling Cell-Based Approach of Pulmonary Artery Smooth Muscle Cells in Response to High Glucose and the Effect of the Antioxidant Vitamins D and E
Source: Metabolites. 2018 Nov 30;8(4):87. doi: 10.3390/metabo8040087 (PMC6316736; doi:10.3390/metabo8040087)
Supplement: Supplementary file 1 [file metabolites-08-00087-s001.pdf]

Supplementary material: Untargeted Metabolic Profiling Cell-Based Approach of Pulmonary Artery Smooth Muscle Cells in Response to High Glucose and Roles of Antioxidant Vitamins D and E.

Abdulwahab Alamri [1,2,*], Abdulhadi S Burzangi [2], Paul Coats [2], David G Watson [2]

[1] Department of Pharmacology College of Pharmacy Sciences University of Hail, 55476 Hail, Saudi Arabia; ph.whab@gmail.com (Alamri, A)

[2] Strathclyde institute of pharmacy and biomedical science, University of Strathclyde, 161 Cathedral street, Glasgow G4 0RE, UK; d.g.watson@strath.ac.uk (Watson, D.); paul.coats@strath.ac.uk (Coats, P.); abdulhadi.burzangi@strath.ac.uk (Burzangi, A.)

[*] Correspondence: ph.whab@gmail.com Tel: +44-141-548-2651; Fax +44-141-552-6443

Table S1: Significantly changed metabolites in PASMCs resulting from treatment with HG or HG + vitamin D or E.

Mass	RT	Met name	-/+	25 mM/ 5 mM glucose		Vit. D/25 mM glucose		Vit. E/25 mM glucose	
				P.value	Ratio	P.value	Ratio	P.value	Ratio
**Glutathione homeostasis**									
230.019	16.7	Xylulose 5-phosphate *	-	<0.001	2.041	<0.001	0.520	0.153	0.726
232.035	16.4	Ribitol 5-phosphate	-	0.017	1.666	0.007	0.393	0.032	0.444
307.084	14.5	Glutathione*	-	0.003	0.801	0.010	1.277	0.023	1.495
335.079	14.5	S-Formyl glutathione	+	0.007	1.290	<0.001	0.234	0.020	0.217
744.076	17.6	NADP+	-	0.031	0.719	0.216	1.156	0.719	1.122
745.085	18.4	NADPH	-	0.532	0.819	0.044	0.323	0.134	0.604
612.152	17.5	Glutathione disulfide *	-	0.025	1.417	0.011	0.606	0.965	1.044
**Purines and pyrimidines**									
111.043	10.4	Cytosine*	-	0.206	1.084	0.004	0.635	0.006	0.679
136.038	10.2	Hypoxanthine *	+	0.009	1.221	<0.001	8.943	0.002	8.221
151.049	11.8	Guanine *	+	0.046	1.675	0.061	0.095	0.005	0.367
152.033	12.0	Xanthine *	-	0.473	1.374	0.603	1.209	0.625	1.225
158.044	14.7	Allantoin	-	0.033	0.535	0.099	0.460	0.132	0.542

168.028	13.6	Urate	-	0.979	0.992	0.127	0.396	0.168	0.502
244.070	9.9	Uridine *	-	0.002	1.584	<0.001	0.297	0.004	0.269
244.070	12.2	Pseudouridine	-	0.017	1.381	<0.001	0.337	0.279	0.408
268.081	11.0	Inosine*	+	0.154	0.381	0.083	4.940	0.173	8.201
324.036	17.4	UMP *	-	0.033	1.580	0.002	0.498	0.004	0.593
332.053	15.4	Deoxyinosine monophosphate	+	0.032	0.598	0.138	1.422	0.300	1.244
347.063	14.3	AMP *	-	0.808	1.019	0.001	0.560	0.074	0.681
348.047	16.2	IMP	-	0.794	0.861	0.861	0.906	0.722	1.201
403.018	18.3	CDP	-	0.836	1.013	0.001	0.592	0.319	0.777
427.029	16.2	ADP *	+	0.699	1.039	0.009	0.718	0.457	0.828
482.985	21.1	CTP	-	0.161	0.894	0.002	0.447	0.089	0.580
483.969	20.2	UTP	-	0.310	0.870	<0.001	0.339	0.005	0.380
506.996	17.1	ATP *	+	0.010	0.858	<0.001	0.476	0.094	0.593
488.108	15.8	CDP-choline	-	0.014	0.694	0.623	1.035	0.151	1.381
537.076	17.6	CDP-ribitol	-	0.009	1.944	0.291	0.429	0.072	0.687
**Glucose metabolism**									
90.032	10.4	(R)-Lactate	-	0.304	1.384	0.004	0.459	0.087	0.581
87.010	8.8	Pyruvate *	-	0.098	1.076	0.431	0.971	0.351	1.147
116.011	15.8	Fumarate *	-	0.023	1.286	<0.001	0.303	0.009	0.429
118.027	16.4	Succinate *	-	0.339	1.191	0.278	0.708	0.496	0.754
134.022	17.4	(S)-Malate *	-	0.034	1.171	0.015	0.660	0.019	0.763
169.989	16.3	Glyceraldehyde 3-phosphate*	-	0.022	5.157	0.397	0.911	0.657	0.889
169.989	17.3	Dihydroxyacetone phosphate*	-	0.001	4.408	0.018	0.816	0.199	0.686
192.019	19.6	Citrate *	-	0.018	0.763	0.272	0.077	0.534	0.006
196.058	14.4	Gluconic acid	-	0.005	1.364	<0.001	0.199	<0.001	0.267
260.030	17.8	Glucose 6-phosphate*	-	0.004	1.230	0.158	0.738	0.279	0.783
260.030	16.8	Glucose 1-phosphate *	-	0.041	2.055	0.017	0.686	0.204	0.792
260.030	17.2	Fructose 6-phosphate*	-	0.009	2.37	0.012	0.81	0.077	1.08
289.033	17.2	Sedoheptulose 7-phosphate	-	0.040	2.119	0.896	0.992	0.671	1.098

318.030	16.4	Octulose 8-phosphate	-	<0.001	1.229	0.008	0.261	0.052	0.436
566.055	17.4	UDP-glucose	-	0.012	1.312	<0.001	0.507	0.056	0.693
580.035	20.7	UDP-glucoronate	-	0.082	1.170	<0.001	0.443	0.028	0.502
663.109	14.4	NAD+ *	-	0.151	1.005	<0.001	0.571	0.636	0.044
665.125	14.1	NADH *	-	0.016	1.177	0.021	0.818	0.385	0.851
666.222	18.0	Maltotetraose	-	<0.001	4.581	0.035	0.824	0.622	0.938
809.120	13.0	Acetyl-CoA *	-	0.165	1.127	0.284	0.702	0.604	0.840
828.275	18.5	Maltopentaose	-	0.053	6.750	0.775	0.979	0.662	1.148
320.051	17.5	Octulose phosphate	-	<0.001	4.441	0.008	1.607	0.152	1.719
350.062	17.7	Nonulose phosphate	-	<0.001	893.963	0.053	1.279	0.097	1.620
**Fatty acids and Lipids**									
101.024	13.3	Acetoacetate *	+	0.007	1.266	0.228	0.924	0.410	0.868
214.193	3.8	Tridecanoic acid	-	0.040	0.805	0.013	1.994	0.021	1.764
216.136	7.4	Undecane dioic acid	-	0.003	0.778	0.074	0.901	0.077	0.894
228.209	3.7	Tetra decanoic acid	-	0.017	0.587	0.016	1.751	0.029	1.809
238.157	3.8	Oxotetradecadienoic acid	-	0.040	1.291	0.482	0.935	0.761	0.973
242.225	3.7	Pentadecanoic acid	-	0.049	0.673	0.018	1.880	0.046	1.739
256.240	3.7	Hexadecanoic acid	-	0.009	0.773	0.143	1.563	0.016	2.111
270.256	3.7	Heptadecanoic acid	-	0.023	0.581	0.005	1.712	0.039	1.941
280.240	3.7	Linoleic acid	-	0.474	1.132	0.024	1.749	0.064	1.843
284.272	3.7	Octadecanoic acid	-	0.021	0.682	0.213	1.547	0.040	2.016
298.287	3.6	Nonadecanoic acid	-	0.005	0.714	0.005	1.727	0.026	2.154
308.272	3.7	Eicosadienoic acid	-	0.911	1.011	0.014	1.630	0.042	1.803
312.303	3.6	Eicosanoic acid	-	0.002	0.666	0.029	1.682	0.035	1.918
332.272	3.6	Docosatetraenoic acid	-	0.009	1.330	<0.001	4.742	0.001	5.234
334.287	3.7	Docosatrienoic acid	-	0.356	1.174	0.001	2.165	0.023	2.771
340.334	3.6	Docosanoic acid	-	0.005	0.686	0.038	1.632	0.145	1.220
366.350	3.6	Nervonic acid	-	0.032	0.774	0.011	0.779	0.169	0.739
767.115	7.5	CoA	-	0.007	0.762	0.154	0.744	0.263	0.725

410.243	4.3	LPA16:0	-	0.047	0.871	0.005	0.788	0.197	0.797
436.259	4.2	LPA18:1	-	0.015	0.828	<0.001	0.584	0.022	0.590
453.285	4.2	LPE16:0	+	0.035	0.747	0.010	0.481	0.029	0.585
479.301	4.2	LPE18:1	-	0.004	0.739	<0.001	0.329	<0.001	0.370
497.275	4.0	LPS16:0	-	0.003	0.713	0.096	0.868	0.330	0.833
523.291	3.9	LPS18:1	-	0.009	0.861	<0.001	0.335	0.009	0.389
525.307	3.9	LPS18:0	-	0.005	0.826	<0.001	0.467	0.016	0.541
545.275	3.9	LPS20:4	-	0.015	1.335	0.723	1.037	0.563	1.127
556.280	3.7	LPG22:6	-	0.022	1.206	<0.001	0.402	0.021	0.516
569.276	3.9	LPS22:6	-	0.042	0.753	0.193	0.186	0.004	0.256
572.296	4.0	LPI16:0	-	0.012	0.852	<0.001	0.262	0.001	0.271
596.296	4.0	LPI18:2	-	0.037	0.754	0.001	0.412	0.021	0.369
600.328	4.0	LPI18:0	-	0.047	0.950	<0.001	0.209	0.002	0.223
620.296	4.0	LPI20:4	-	0.011	0.874	<0.001	0.482	0.032	0.514
698.489	3.8	PA36:3	-	0.001	0.732	0.001	0.579	<0.001	0.430
700.505	3.7	PA36:2	-	0.007	0.769	0.046	0.852	0.001	0.588
720.494	3.5	PG32:1	-	0.026	1.539	0.044	0.843	0.293	0.809
731.546	3.8	PC32:1	-	0.897	0.917	0.001	0.429	0.001	0.355
755.546	3.7	PC34:3	-	0.028	0.670	0.078	0.143	0.082	0.160
779.546	3.8	PC36:5	-	0.659	1.064	0.001	0.394	0.001	0.404
783.505	3.5	PS36:4	-	0.041	1.308	0.007	1.586	0.133	1.176
805.561	3.7	PC38:6	-	0.810	1.019	<0.001	0.448	<0.001	0.404
820.526	3.4	PG40:7	-	0.001	0.688	<0.001	0.175	<0.001	0.176
846.542	3.4	PG42:8	-	0.021	0.805	0.020	0.810	0.105	0.700
856.510	3.5	PI36:5	-	0.028	0.627	0.289	0.382	0.004	0.382
**Carnitines**									
231.147	8.4	Butyrylcarnitine	-	0.956	0.994	<0.001	0.002	<0.001	0.002
245.162	5.2	Valerylcarnitine	-	0.217	1.012	<0.001	0.001	<0.001	0.001
397.319	4.3	Hexadecenoylcarnitine	+	0.036	1.336	0.017	0.687	0.143	0.828

413.314	4.5	Hydroxyhexadecenoylcarnitine	+	0.374	0.003	<0.001	1654	<0.001	1637
425.350	4.3	Oleoylcarnitine	+	0.018	1.283	0.006	0.500	0.023	0.547
**Amino acids**									
103.063	15.7	Aminobutanoate	+	0.030	1.194	0.001	1.410	0.038	1.635
117.079	12.2	Aminopentanoate	+	0.015	1.213	<0.001	0.271	0.004	0.339
146.106	25.2	Lysine*	+	0.044	1.133	0.091	0.866	<0.001	0.412
218.127	14.4	N2-(D-1-Carboxyethyl)-L-lysine	+	0.002	1.535	<0.001	0.240	0.005	0.336
119.058	15.0	Threonine*	-	0.626	1.085	0.009	0.556	0.049	0.591
125.014	15.9	Taurine*	-	0.058	1.107	<0.001	0.227	0.002	0.276
132.053	15.9	Asparagine	+	0.074	0.798	<0.001	0.216	0.001	0.240
132.090	23.8	Ornithine	+	0.549	0.988	0.021	0.365	0.026	0.379
133.037	15.8	Aspartate *	-	0.007	0.707	<0.001	0.289	0.004	0.374
147.050	15.4	Glutamate*	-	0.024	1.453	0.078	0.83	0.154	1.10
89.047	15.2	Alanine*	-	0.482	1.108	<0.001	0.258	0.001	0.279
204.090	11.8	Tryptophan*	-	0.008	1.152	0.059	0.852	0.112	0.471
174.112	26.6	Arginine*	-	0.035	1.141	0.020	0.861	0.002	0.723
115.063	12.9	Proline*	+	0.001	1.212	0.036	0.936	0.131	1.052
161.069	15.6	N-Methyl-L-glutamate	+	0.021	1.182	<0.001	0.390	0.004	0.386
131.069	14.9	Creatine*	-	0.925	0.965	0.001	0.374	0.005	0.488
175.100	16.1	Citrulline*	-	<0.001	0.832	0.011	0.87	0.002	0.55
155.070	15.8	Histidine*	-	0.004	0.774	0.632	0.94	0.087	0.71

(*) means that metabolite matches standard retention time

Diagram below illustrates the steps of data filtration:

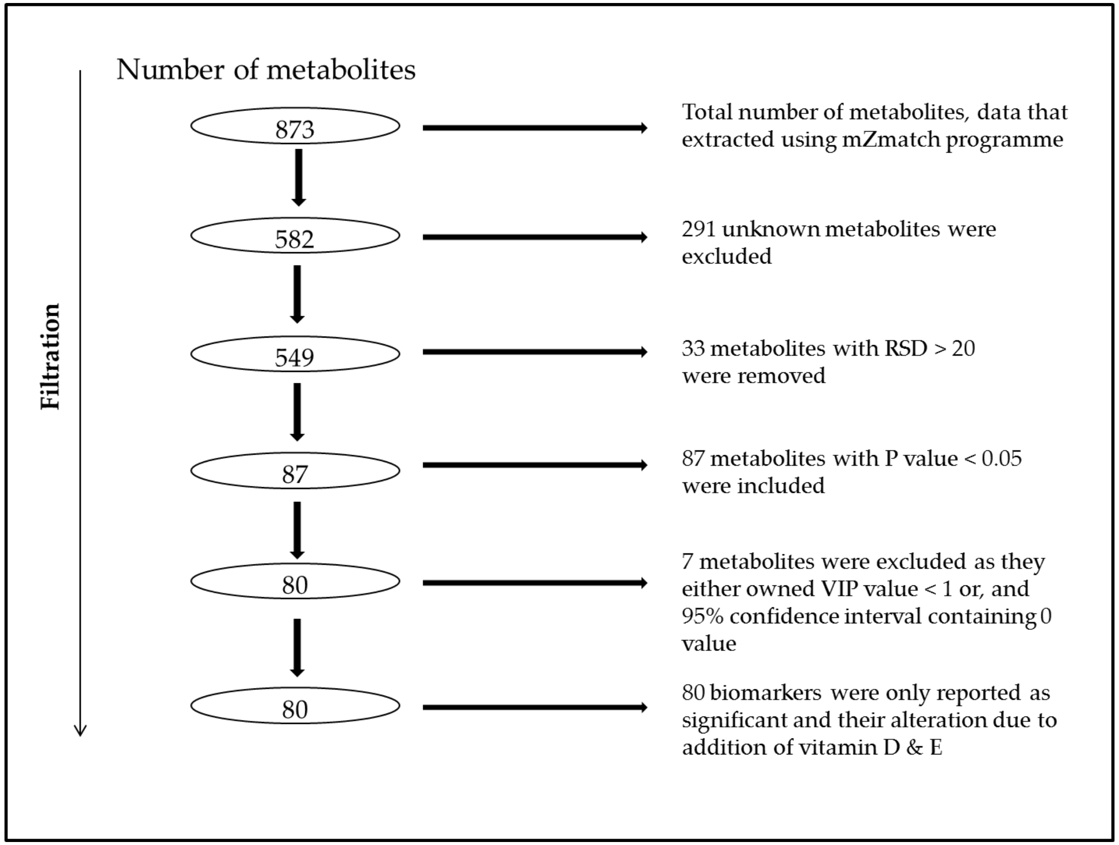

Figure S1: Data filtration steps.

Extracted data were normalized using log2 transformation, how data distribution has changed can be seen in the figure below.

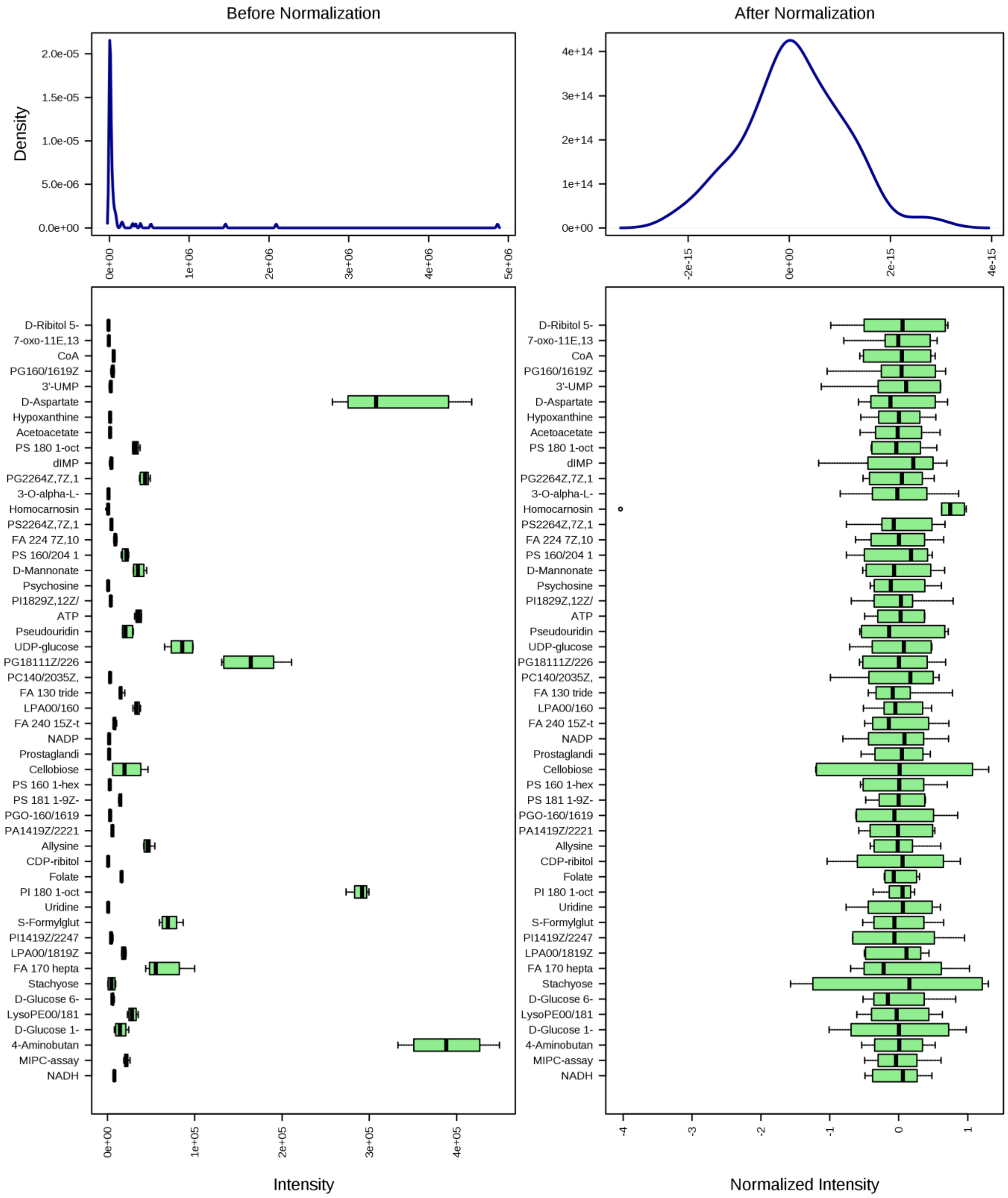

Figure S2: How data log_2 transformation of metabolites' intensity improves data distribution.

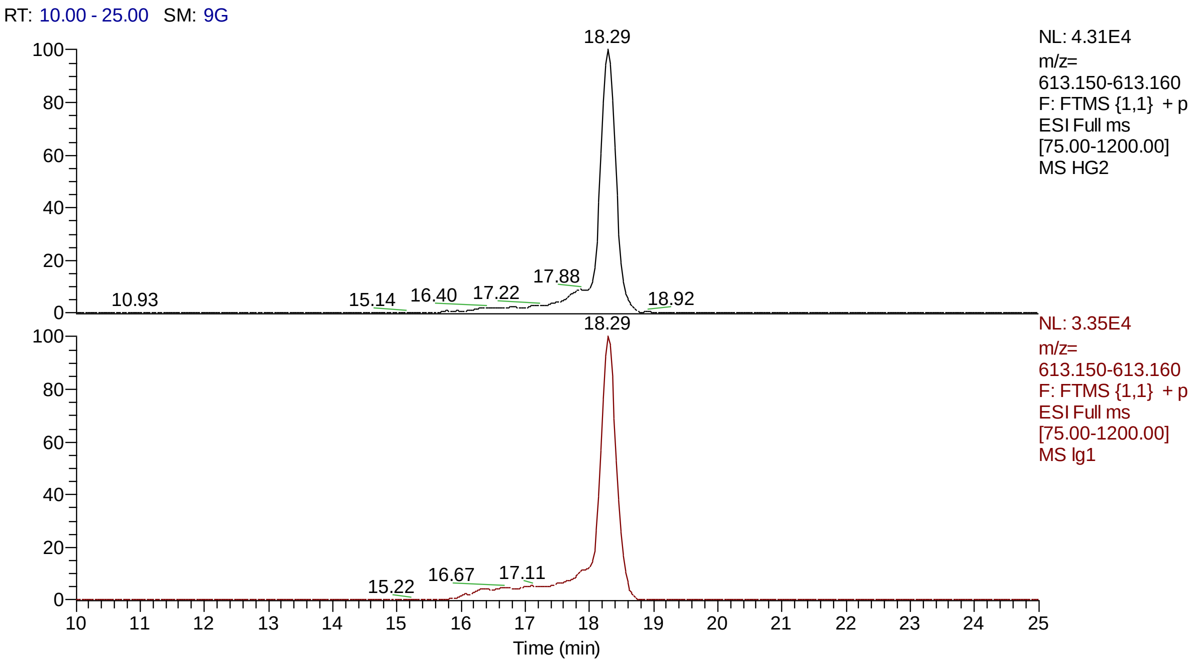

Figure S3: Comparison of extracted ion traces for GSSG, HG media, and LG media.

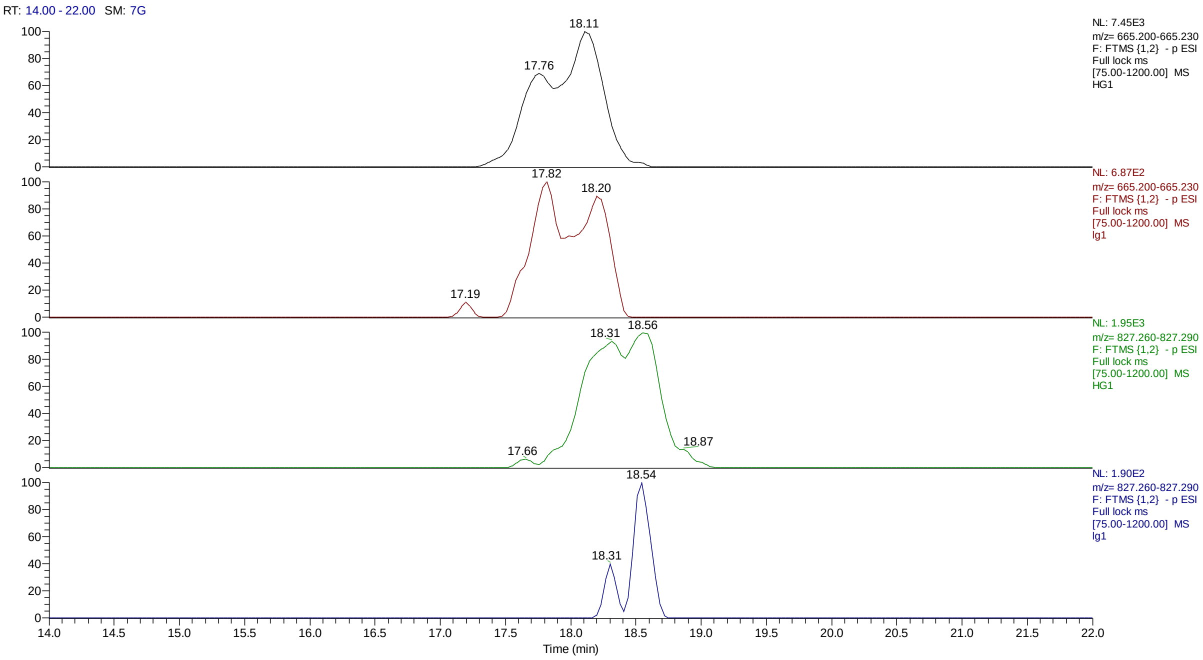

Figure S4: Extracted ion traces for maltotetraose and maltopentaose in HG and LG media.
